# Heritable determinants of male fertilization success in the nematode *Caenorhabditis elegans*

**DOI:** 10.1186/1471-2148-11-99

**Published:** 2011-04-14

**Authors:** Rosalind L Murray, Joanna L Kozlowska, Asher D Cutter

**Affiliations:** 1 Department of Ecology and Evolutionary Biology, University of Toronto, Toronto, M5S 3B2, Ontario, Canada; 2 School of Biological and Environmental Sciences, University of Stirling, Stirling, FK9 4LA, UK

## Abstract

**Background:**

Sperm competition is a driving force in the evolution of male sperm characteristics in many species. In the nematode *Caenorhabditis elegans*, larger male sperm evolve under experimentally increased sperm competition and larger male sperm outcompete smaller hermaphrodite sperm for fertilization within the hermaphrodite reproductive tract. To further elucidate the relative importance of sperm-related traits that contribute to differential reproductive success among males, we quantified within- and among-strain variation in sperm traits (size, rate of production, number transferred, competitive ability) for seven male genetic backgrounds known previously to differ with respect to some sperm traits. We also quantified male mating ability in assays for rates of courtship and successful copulation, and then assessed the roles of these pre- and post-mating traits in first- and second-male fertilization success.

**Results:**

We document significant variation in courtship ability, mating ability, sperm size and sperm production rate. Sperm size and production rate were strong indicators of early fertilization success for males that mated second, but male genetic backgrounds conferring faster sperm production make smaller sperm, despite virgin males of all genetic backgrounds transferring indistinguishable numbers of sperm to mating partners.

**Conclusions:**

We have demonstrated that sperm size and the rate of sperm production represent dominant factors in determining male fertilization success and that *C. elegans *harbors substantial heritable variation for traits contributing to male reproductive success. *C. elegans *provides a powerful, tractable system for studying sexual selection and for dissecting the genetic basis and evolution of reproduction-related traits.

## Background

Anisogamy, the occurrence of different sized gametes in different mating types or sexes, commonly manifests as small male gametes and large female gametes; the small male gametes (sperm) tend to be more numerous than female gametes (oocytes) [1]. When two or more males compete for the fertilization of oocytes in a multiply-mated female, then it is often true that the male that produces the most sperm will procure the greatest fertilization success [1]. This type of sperm competition (a 'fair raffle') can lead to selection for more, and further miniaturized, male gametes as limited resources are allocated to create more individual gametes [2,3]. However, if multiple sperm actively and directly compete for fertilization rather than being used passively in such a 'lottery,' then the evolution of larger sperm size commonly evolves [4-7] - potentially at the expense of ejaculates containing fewer sperm. Thus, sperm number per ejaculate and sperm size form two important components affecting post-mating fertilization success, with potentially differing fitness optima and developmental constraints that depend on the details of the regime of sperm competition.

Polyandrous mating behavior inducing sperm competition can cause antagonistic co-evolution between the sexes [8-10] and manifest as a suite of traits that includes copulatory plugs [11-13], oocyte stimulation [14], mate guarding [15,16] and sperm expulsion by females [13,17]. Some or all of these traits are common across a wide range of taxa, but their relative importance with respect to sperm competition can be difficult to decipher. Here, we investigate male-male sperm competitive ability in the nematode model organism *Caenorhabditis elegans *to better understand the relative importance of mating and sperm traits for heritable variation in male reproductive success.

*Caenorhabditis elegans *is an androdioecious species that consists of males and self-fertilizing hermaphrodites, but evolved from gonochoristic (male/female) ancestors in the relatively recent past [18-20]. Males are rare in nature, but can successfully mate with hermaphrodites when given the chance [reviewed in [21,22]]. Males also make larger sperm than hermaphrodites, and male sperm are used preferentially over self-sperm for fertilization in a mated hermaphrodite [23,24]. While perhaps not common for *C. elegans *in nature, male-male sperm competition likely is an important aspect of mating system evolution in closely related gonochoristic species that retain the ancestral mode of reproduction. With the extensive genetic and developmental tools available for *C. elegans*, this species provides an exceptionally tractable model system to address general questions about the evolution of sperm traits, as well as more specific issues pertinent to *Caenorhabditis *mating systems. For example, LaMunyon and Ward [25] demonstrated how laboratory-induced sexual selection caused the evolution of larger male sperm, and a variety of alternative regimens of experimental evolution have explored the evolution of hermaphrodite sperm production, sex-ratio and outcrossing rate [reviewed in [21,26,27]].

Several traits have identified themselves as being individually important to male fertilization success in *C. elegans*. As in many animals, mating rate is important [28], such that males capable of mating often and repeatedly have higher paternity [29,30]. Similarly, we expect that the number of sperm that a male passes to a hermaphrodite during a single mating event likely will be important for sperm competition success, in addition to the number of matings - as in domestic fowl (*Gallus gallus domesticus*) [31] and golden egg bugs (*Phyllomorpha laciniata*) [32]. However, we know of no direct tests for such an effect in *Caenorhabditis*. In other systems, the duration of copulation directly affects the number of sperm that a male passes to a female in a given mating bout [33] and the more sperm he passes, the greater his success in sperm competition [34]. The role of hermaphrodites in attracting (or avoiding) potential mates also likely affects male mating rate and could influence male postcopulatory competitive ability [21]; as an extreme case, male *C. elegans *mate more readily with individuals that are motility defective [35]. It is now clear that *C. elegans *hermaphrodites have lost the ability to produce potent attractive pheromones to attract mates [36], despite males tending to spend more time on media that has been occupied previously by a hermaphrodite [37-39]. *C. elegans *males, however, have not lost the ability to detect and seek out females from related species like *C. remanei *that do release attractive pheromones [36,40], although *C. elegans *male mating ability is poorer than that of *C. remanei *males [35]. Despite the importance of seminal fluid [41] and male age [42,43] in other systems, they do not seem to influence sperm competitive ability in *C. elegans *[24]. Perhaps most-studied in terms of the influence on male sperm competition in *C. elegans *is the effect of sperm size on paternity: male sperm are larger than hermaphrodite sperm and male sperm are used preferentially for fertilization [23,44,45]. Males of two genetically distinct strains of *C. elegans *make differently-sized sperm, and the larger sperm have greater sperm precedence, although they take longer to make [44]. And yet, we don't know whether there might be a trade-off between the ability of males to transfer sperm that individually are highly competitive (i.e. large sperm) with their ability to transfer many sperm. Moreover, most studies on these issues have focused on strains with the standard N2 genetic background, which are notoriously poor at mating and have small sperm [21].

In order to capture a deeper understanding of the forces contributing to the evolution of reproductive systems, here we test the relative importance of a range of mating and sperm traits on siring success with a diverse set of distinct genetic backgrounds. Male genotypes were chosen based on known differences in genetic composition, whether or not they produced a copulatory plug, and when possible, male maintenance and mating ability. We hypothesize that genotypes with greatest male reproductive success will map to phenotypes that include large sperm that are produced quickly to be transferred in sperm-dense ejaculates, coupled with high courtship and mating rates and greater sperm precedence.

## Methods

### Culturing and Maintenance

We obtained all strains used in this experiment (Table 1) from the Caenorhabditis Genetics Center (CGC), and cultured and maintained the nematodes in Petri plates on NGM-Lite agar seeded with *E. coli *strain OP50 at 20°C [46,47]. Strains were chosen to maximize diversity along several dimensions, including being genetically distinct, presence/absence of a copulatory plug after mating, and, when available, information about their ability to maintain males in a population, and male mating ability. Strains represent essentially isogenic lines, so significant differences among them are directly attributable to heritable differences.

**Table 1:** Strains used, their geographic location of origin or mutation and relevant notes*

Type	Strain	Origin	Notes
Wild Isolates	AB1	Adelaide, Australia	no plug, 'large-sperm' category
	CB4855	Stanford, USA	plug, 'large-sperm' category
	CB4856	Hawaii, USA	plug, 'large-sperm' category
	DR1350	Pasadena, USA	plug, 'small-sperm' category
	JU440	France	no plug, 'small-sperm' category
	MY2	Germany	no plug, 'small-sperm' category

Experimental Mutants	JK574	*fog-2(q71) *mutation in an N2 genetic background	Populations 50% male + 50% "female"
	PD4790	contains transgene *mls12 [myo-2::GFP, pes-10::GFP, F22B7.9::GFP] *in an N2 genetic background	N2 background with pharyngeal GFP marker, no plug, 'small-sperm' category

*Strains grouped into sperm size categories based on the results of Tukey HSD test where 'small-sperm' strains were not significantly different from one another.

### Sperm size measurements

**Figure 1:**
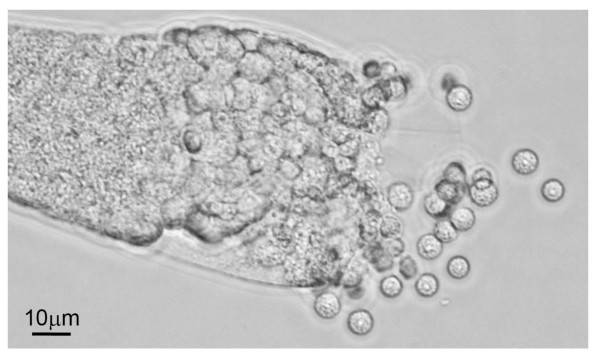
**Dissected gonad of a male *C. elegans *of strain CB4856**. Spermatids (immature, round sperm cells) are being released from the gonad into Sperm Medium buffer solution.

We measured male sperm size as spermatid diameter for seven strains of *C. elegans *based on the method of LaMunyon and Ward [45]. Nematode spermatids are haploid immature gametes that, upon maturation into spermatozoa, sprout a pseudopod that allows motility within the female or hermaphrodite reproductive tract. Despite the irregular shape of spermatozoa, the cell body retains the same volume as the spermatids [48] which are generally spherical and, thus, relatively easy to measure. Male worms were standardized for age by collecting them as last-stage juveniles (L4) and isolating them for ~20 hours before they molted to adulthood, to ensure virginity. *C. elegans *males store their sperm as spermatids in the gonad. We dissected out male gonads in Sperm Medium [49] and viewed and photographed the released spermatids under DIC optics with a 40× objective lens (Figure 1). The cross-sectional area (A) and volume (V) of the spermatids was estimated from measures of diameter (D) as A = πr^2 ^and V = 4/3 πr^3 ^where cell radius r = D/2, using the imaging software ImageJ version 1.42q on digital photographs. We report analyses of spermatid diameter only; area and volume metrics are provided in Additional File 1 to facilitate comparison with other studies [e.g., [25,45]]. An average of 106 spermatids were measured from each strain (range 95-122, based on 7-11 males of a given strain); we treated spermatids as the unit of replication in analysis because dissection of multiple males on a slide meant that spermatids could not be associated confidently with particular individuals.

### Rate of sperm production

We measured the rate of sperm production in virgin males, modeled after LaMunyon and Ward [44]. L4 male worms were monitored in 15-minute intervals for the molt to adulthood, and if a worm was newly molted we either fixed and stained it with DAPI nucleotide stain immediately or isolated it for 2 hours prior to fixation and staining. We then counted sperm numbers for 16-21 males per strain for each time-point by identifying DAPI-stained spermatid nuclei [50]. DAPI-labeled worms were mounted on a glass slide so that sperm nuclei could be viewed under epifluorescence and counted from digital photographs taken in different focal planes through the specimen.

### Number of sperm transferred

We quantified the distribution of transferred sperm counts from male ejaculates to test for association with other sperm traits. First, we isolated 10 *fog-2 *females (strain JK574) as L4s one day in advance of the assay to ensure that they were virgins. The *fog-2 (q71) *allele that is homozygous in the JK574 strain affects the sperm-oocyte switch of the hermaphrodite ovotestis, such that hermaphrodites are capable of making only oocytes [51]; hence, we refer to such individuals as "females," which must mate with males to reproduce. Similarly, we also separated males onto plates as L4s from each of the seven experimental strains (AB1, CB4855, CB4856, DR1350, JU440, MY2) and the reference strain (PD4790). The next day, we began the assay by adding individual males to each of the plates containing the 10 females. This female-biased sex ratio ensures that males are unlikely to mate with the same female more than once. We inspected each plate every hour thereafter, and as soon as fertilized eggs were observed, we separated out each female onto an individual plate and discarded the male. If there were no eggs laid by the end of twelve hours, we discarded the plate. The day following the matings, we scored females as either mated or not mated based on the presence or absence of eggs on the plate. We transferred mated females onto new plates daily until they ceased egg production. The resulting progeny were counted when they reached the L4 or young adult stage. For a given mated female, the total progeny count provides our measure of the number of sperm in a single ejaculate. If more than one female was mated by a male, the number of eggs on the initial mating plate was divided evenly between them. Given the highly female-biased sex ratio, generally poor mating-ability of *C. elegans *males, and frequent monitoring, we assume that a mated female was inseminated only once. The measures for the number of sperm transferred are not downwardly-biased by female fecundity, because females are capable of producing many times more oocytes when mated *ad libitum *relative to this assay and sperm are highly efficient at achieving fertilization upon insemination [23]. Plates were maintained at 20°C throughout the experiment. We assayed 10 to 21 individuals per strain.

### Male mating ability

Following the "9-hour assay" of Wegewitz et al. [30], we isolated 14 L4 *fog-2 *"females" (strain JK574) per plate one day prior to the start of the experiment to ensure their virginity. We also isolated multiple L4 males per plate, without hermaphrodites, from each experimental strain (AB1, CB4855, CB4856, DR1350, JU440, MY2) and the reference strain (PD4790) the day before the experiment. To begin the assay, we transferred a single male onto a mating plate with the 14 virgin hermaphrodites. Over the next 9 hours, we inspected the males 14 times (after 10 min, 1 h, 2 h, 4 h, and every 30 min thereafter). At every inspection, we scored the male as: (i) in no contact with any females, (ii) in contact with a female, or (iii) with spicules inserted in copulation. Because males were rarely observed *in copula*, we analyzed (ii) + (iii) in combination. Following the 9-hour assay, we isolated each female; we then scored them the following day as either mated or not mated, based on the presence of eggs or young larvae on the plate, as our measure of copulatory success that resulted in sperm transfer. We performed this assay on 10 to 13 males per strain. Following our ANOVA analysis of these data, we performed post-hoc comparisons comparing strains with the minimum (Hsu's MCB), rather than all possible pairwise comparisons.

### Male-male sperm competition (P_1 _and P_2_)

We measured the first- (P_1_) and second-male sperm precedence (P_2_) of the six wild isolate *C. elegans *strains in sperm competition with a reference strain. "Females" from strain JK574 were mated sequentially to two males: a PD4790 reference strain marked phenotypically with the genetically dominant, pharyngially-expressed green fluorescent protein (GFP), and a male from one of six wild isolate strains (Table 1). We changed the mating order of the rival males of different strains as either first or second mates. Excepting the lab-derived allele *fog-2 (q71) *and transgene *mls12 (myo-2::GFP, pes-10::GFP, F22B7.9::GFP)*, the genetic background of JK574 and PD4790 is identical; both are derived from the canonical strain N2.

Males and females were isolated in the last larval stage and maintained as virgins for 24-30 hours prior to mating trials. Isolated males or females that crawled off the media and onto the sides of plates were either rescued, and included in the study, or died and were excluded. We then placed a single virgin female on a plate for 4 hours with 8 males of a given strain. The males were then removed and replaced with 8 males from a different strain for another 4 hours. Thus, a given female was mated sequentially to one or more males from each of two strains: reference strain PD4790 and a wild isolate strain. In this way, the sperm from different male genotypes is placed in direct competition within the female's reproductive tract. Following both matings, the females were placed on a new plate where they continued to lay eggs. Eggs laid in the mating arena during the 4-hour mate-access periods were discarded. Females were subsequently transferred to a new plate after 18 hours, and then again 24 hours later to time-stamp the progeny as early (first 18 hours after second mating) or late (any eggs laid after that).

A total of 111 females were mated to males. Eight females died before they had laid all of their eggs and were excluded from the dataset. Of the remaining 103, 14 showed complete sperm precedence for one strain indicating that one male genotype failed to mate successfully during the trial, and also were excluded (9 indicated no sperm transferred during the second mating, and 5 indicated no sperm transferred during the first mating). In all cases, reference strain PD4790 was the strain that failed to mate successfully, which corroborates the relatively poor male mating ability of the N2 genetic background [29,30]. Paternity (P1 or P2, early or late) was assigned on the basis of GFP phenotype, with adult progeny scored as either GFP (sired by reference PD4790 males) or non-GFP (sired by one of the six wild isolate males). There were between 14 and 16 successful sperm competition assays per strain combination.

### Integrating Male Reproductive Traits

We used principle components (PC) analysis to explore inter-relationships among trait means (or medians, for traits with skewed distributions) for the different genetic backgrounds. We implemented the PC analysis in JMP, including values for the following traits for each of the 7 strains (Table 2): courtship ability, mating ability, number of sperm transferred, spermatid size, sperm production rate, P_2-early _and P_2-late_. We also calculated pairwise correlations among average trait values for the *C. elegans *genetic backgrounds to provide another view of trait associations. However, there is limited power to infer significant effects with the 7 genetic backgrounds assayed in this study, so we present the correlations only in Additional File 2.

**Table 2:** Summary of male reproductive traits.

strain	courtship ability ^a^*	mating ability ^b^*	number of transferred sperm ^c^	spermatid size ^d^*	sperm production rate ^e^*	P2 early*	P2 late	P2 fertility ^f^	male maintenance ^g^
AB1	23.38	12.14	161	8.17	0.780	0.734	0.490	190.2	0.352
CB4855	29.17	8.57	188	5.62	0.708	0.814	0.516	266.2	0.122
CB4856	27.14	8.57	178	6.08	0.758	0.809	0.495	260.3	0.239
DR1350	9.52	5.00	206	5.15	1.017	0.497	0.519	196.3	n.d.
JU440	20.83	23.57	111	5.46	0.935	0.572	0.500	216.7	0.050
MY2	22.53	20.00	138	4.22	0.862	0.516	0.460	159.5	0.135
PD4790	24.03	18.57	123	4.74	0.970	0.488 ^h^	0.496 ^h^	170.1	0.181 ^i^

^a ^mean percent of observations with male-female contact and/or spicule insertion; ^b ^mean percent females inseminated in 9 h; ^c ^median number of sperm; ^d ^median diameter (μm); ^e ^mean number of sperm/min; ^f ^mean total number of progeny; ^g ^from Teotonio et al. [29]; ^h ^averaged across all 6 experiments with wild isolate strains; ^i ^value for strain N2 that shares the same genetic background as PD4790; * significant among-strain heritable variation in this trait; n.d. = not determined

## Results

### Heritable variation in male sperm traits

**Figure 2:**
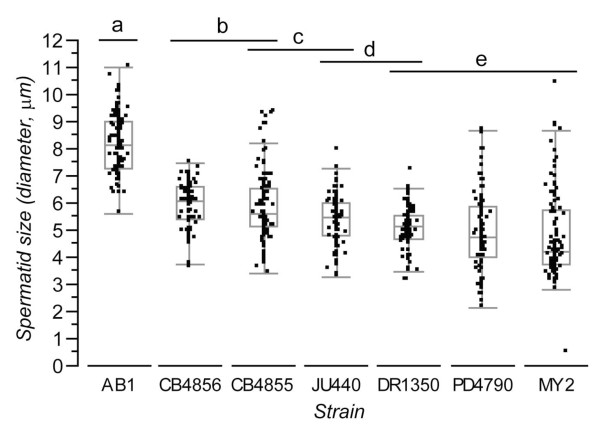
**Size distributions of male spermatids for each of 7 strains (diameter, μm)**. Box-plots indicate median and inter-quartile range (points beyond whiskers are candidate outlier values). Horizontal bars at the top indicate Tukey HSD groups, with strains sharing identical letters not differing significantly.

We measured sperm traits for males from seven isogenic strains of *C. elegans *and found that several traits differ significantly among them. First, strains differed significantly in male sperm size, using any of three measures of spermatid size (diameter, area or volume; Figure 1, Figure 2, Additional File 1). For brevity, here we focus statistics and figures on values for spermatid diameter. Inter-strain differences account for 46% of the variation in spermatid size (one-way ANOVA F_6,735 _= 104.4, P < 0.0001). Post-hoc tests (Tukey HSD) indicate that AB1 males have significantly larger sperm than all other strains, and that sperm from CB4856 and CB4855 males are significantly larger than those of DR1350, MY2 and PD4790, while sperm from CB4856 additionally are significantly larger than JU440 (Table 2, Figure 2). These sperm size patterns are consistent with a previous report for four of the genetic backgrounds [25]. In addition, within-strain male spermatid size varied by at least a factor of two (Figure 2). Thus, we identified substantial heritable and non-heritable variation in male sperm size among these seven strains of *C. elegans*.

**Figure 3:**
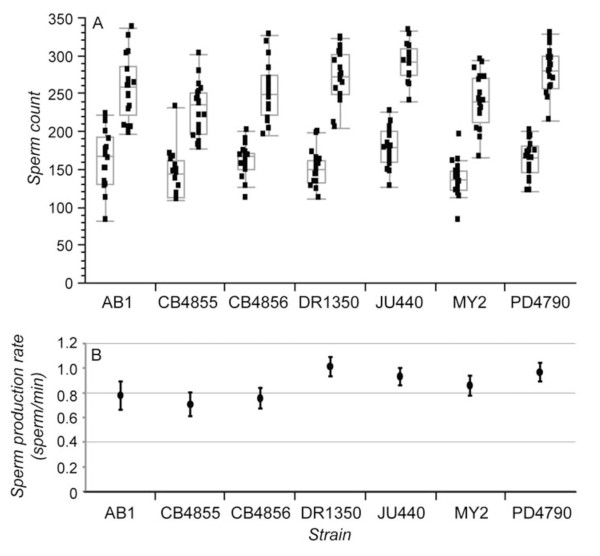
**Sperm production rates for different *C. elegans *male genetic backgrounds**. (A) Box-plots for spermatid counts for each strain at time zero (left) and two hours later (right), indicating median and inter-quartile range (points beyond whiskers are candidate outlier values). (B) Estimates of sperm production rate for each strain, based on the linear regression of sperm counts between the two time periods. Error bars in (B) indicate 1 S.E. for the slope of the regression line estimates of sperm production rate.

We also tested for heritable variation in the rate of sperm production by virgin males from each of the seven isogenic strains (Table 2, Figure 3). When we first tested for heterogeneity in sperm production rates among all strains with ANOVA (factors: strain, timepoint, and strain × timepoint interaction), we detected no significant heterogeneity (interaction between strain and time: F_6, 236 _= 1.82, P = 0.096). However, when we grouped strains based on sperm size (Table 1) and compared three strains with sperm > 5.5 μm median diameter ('large-sperm') to four strains with < 5.5 μm median diameter ('small-sperm'), we found that these two categories differed significantly in their rates of sperm production (ANCOVA factors: sperm size class, timepoint, and sperm size class × timepoint interaction; interaction term F_1, 246 _= 8.38, P = 0.004). Specifically, the strains with larger sperm make them at a slower pace, consistent with a previous comparison of two strains [44].

**Figure 4:**
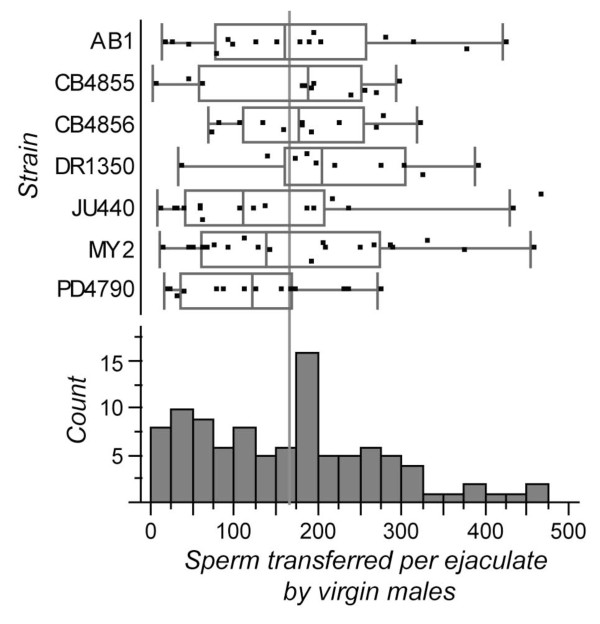
**Distribution of the number of sperm transferred in a single ejaculate by virgin males to females (assayed by female fecundity)**. Box-plots indicate median and inter-quartile range (points beyond whiskers are candidate outlier values). Histogram shows all seven strains combined (no significant differences among strains), with vertical gray bar indicating the grand mean (166.93).

Finally, we tested for an effect of genetic background on the number of sperm transferred per ejaculate by virgin males as assayed by female fecundity. We detected no significant heritable variation for the number of sperm transferred per ejaculate among the seven strains, with males transferring a median of 169 sperm across all strains (one-way ANOVA F_6,94 _= 0.90, P = 0.5) (Figure 4). Note that male gonads contained an average of 227 to 290 spermatids at the second time-point in our sperm production rate assays, implying that males may transfer roughly two-thirds of the spermatids in their gonad. However, dissection of several non-virgin males resulted in none or very few spermatids. Consequently, it is possible that males transfer their entire load of spermatids in each ejaculate, with roughly one-third of their sperm typically getting lost by spillage, passively lost from the female gonad (e.g. by passage of eggs), actively extruded by females, or being otherwise inviable or incompatible [17].

### Male mating ability

**Figure 5:**
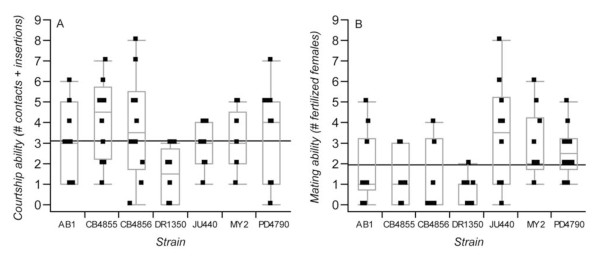
**Male courtship and mating ability for different *C. elegans *strains**. (A) Male courtship was assayed as the number of body contacts and spicule insertions observed with females across 14 point-observations over a 9 hour period in mating arenas comprised of 14 females and a single male. (B) Mating ability was assayed as the number of females that were successfully fertilized in the courtship assay. Box-plots indicate the median and interquartile range (points beyond whiskers are candidate outlier values); horizontal lines indicate the grand mean across strains.

To explore how male mating behavior varies in *C. elegans *and could influence relative siring success, we tested for heritable differences in the frequency of encounters and matings in non-competitive copulatory success (sperm transfer and fertilization). Using the "9-hour assay" developed by Wegewitz et al. [30], we identified significant variation among genetic backgrounds in male courtship (contacts and/or spicule insertions with mates) (one-way ANOVA F_6,74 _= 2.28, P = 0.0158) as well as heritable variation in the number of mates successfully inseminated (one-way ANOVA F_6,63 _= 3.46, P = 0.0051; Table 2, Figure 5). Post-hoc tests (Hsu's MCB) showed that strains JU440, MY2 and PD4790 all had significantly higher mating success than the strain with poorest mating success (DR1350); strain DR1350 also exhibited the lowest courtship ability, with other strains having similar scores in our assay (Table 2, Figure 5).

### Competitive ability of male sperm

**Figure 6:**
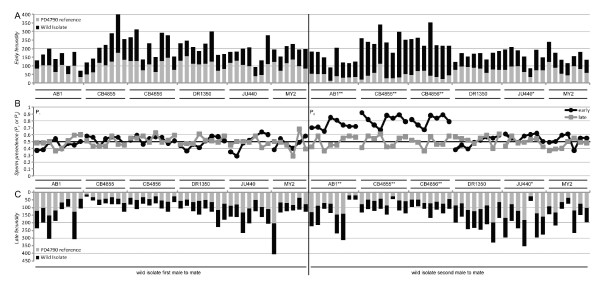
**Sperm precedence of wild isolate male sperm in competition with reference strain PD4790**. (A) Early fecundity for each replicate double-mating (first 18 hours following the second mating), shown cumulatively for the reference strain and the wild isolate. (B) The fraction of offspring sired by wild isolate males when mated to females first (P_1_) or second (P_2_), for early and late time periods. (C) Late fecundity for each replicate double-mating (any offspring laid after the first 18 hours) periods post-mating. Early versus late sperm precedence was statistically tested by paired t-test (AB1, CB4855, CB4856: **, P < 0.0001; JU440: *, P < 0.05; all other pairs not significant).

We also quantified variation among male genotypes in sperm competitive ability. We assayed paternity for males that mated second (P_2_) for progeny produced in two time intervals following the sequential mating period; early (within 18 hours of completion of the second mating) and late (any eggs laid after the early period; Figure 6). We expected that strains with larger sperm will sire a higher proportion of the progeny in the early time period due to preferential use of their larger sperm in fertilization. Consequently, we expected males from strains with large sperm to have higher P_1 _and P_2 _in the early period than males from strains with smaller sperm. We saw that strains with the largest sperm do tend to experience high paternity in the early time period when mated second: strains AB1, CB4855, CB4856 and JU440 all have significantly higher P_2 _in the early time period compared to the corresponding later time period when mated second (Table 2, Figure 6). However, P_1 _early and late values and P_2 _late values did not differ from an equal siring of progeny (50%; Figure 6). We found no clear distinction in sperm precedence between the 3 strains in which males produce a copulatory plug (CB4855, CB4856, DR1350) and the other non-plugging strains.

### Integrating Traits Contributing to Male Reproductive Success

We performed a Principal Components Analysis of the sperm and mating traits using mean or median values for each of the 7 genetic backgrounds to explore the inter-relationships among the factors contributing to male reproductive success. The first two principal component (PC) axes together explain 78% of the variation among strains (43.4% and 34.5%, respectively), with the third and fourth axes contributing an additional 10.8% and 9.3%, respectively (Table 3). The first PC axis is composed of high P_2-early_, slow sperm production, large sperm size, frequent rates of encounter, and low mating rate (Table 3). The second PC axis includes ejaculates with many sperm, low mating rates, low encounter rates, and high P_2-late _(Table 3). The leading factor for PC3 is P_2-late_, and for PC4 is sperm size. We also performed non-parametric pairwise correlations among the trait averages for these strains (Additional File 2), although statistical power is greatly limited by strain number in this analysis.

**Table 3:** Summary of the leading four eigenvectors from a Principal Components (PC) Analysis of male reproduction traits

		factor						
		
PC axis	eigenvalue (%)	**courtship ability **^**a**^	**mating ability **^**b**^	**number of transferred sperm **^**c**^	**spermatid size **^**d**^	**sperm production rate **^**e**^	P2 early	P2 late
PC1	3.04 (43.4%)	0.373	-0.292	0.076	0.395	-0.531	0.566	0.110
PC2	2.41 (34.5%)	-0.454	-0.513	0.553	0.075	0.171	-0.016	0.437
PC3	0.753 (10.8%)	0.129	0.060	-0.540	0.036	0.216	0.040	0.799
PC4	0.654 (9.3%)	-0.307	0.230	-0.115	0.884	0.189	-0.078	-0.132

^a ^mean percent of observations with male-female contact and/or spicule insertion; ^b ^mean percent females inseminated in 9 h; ^c ^median number of sperm; ^d ^median diameter (μm); ^e ^mean number of sperm/min.

## Discussion

Our assays of pre- and post-mating traits demonstrate heritable natural variation for male mating ability, sperm size, rate of sperm production and sperm precedence in *C. elegans*. The number of sperm transferred in a single ejaculate by virgin males, however, does not vary significantly among male genetic backgrounds. We confirm that sperm size is an important indicator of fertilization success in male-male sperm competition and that large sperm come at a cost because they take longer to produce [44] - indeed, we demonstrate that heritable differences in the rate of sperm production is the strongest correlate of second-male sperm precedence.

### The factors contributing to sperm precedence and male reproductive success

The *C. elegans *literature shows that, in mated hermaphrodites, male sperm outcompete self-sperm [23,24] and the larger size of male sperm likely contributes to their superior competitive ability [44]. Following sperm transfer to a hermaphrodite, a *C. elegans *male's amoeboid sperm must crawl up one of two gonad arms to reach the spermathecae (the sites of fertilization), where they can compete with the accumulated self-sperm for access to oocytes. Mature oocytes pass through the spermathecae where they are fertilized, and then into the uterus before exiting the animal through the vulva. During this process, sperm can be carried with the egg as it moves away from the site of fertilization. Sperm in the spermathecae of mated hermaphrodites are significantly larger than sperm in the uterus, indicating that smaller sperm are more likely to be displaced or less likely to re-migrate to the spermathecae following displacement [44]. Displaced sperm risk being expelled to the external environment when an egg is laid. Almost all of a hermaphrodite's relatively small self-sperm can be lost during egg-laying if a hermaphrodite receives enough male sperm [23]. Thus, being able to crawl faster back into the spermathecae or being able to adhere better to the reproductive tract likely are beneficial sperm traits; both have been observed *in vitro *as characteristic of larger sperm [44]. These characteristics must be critical components of sexual selection by male-male sperm competition in nature for gonochoristic relatives of *C. elegans*.

In a direct test of inter-male sperm competitive ability, we saw that genetically distinct strains with larger sperm had greater early paternity when mated second (high P_2-early_). This indicates that when the male with larger sperm is mated second to a *fog-2 *female, the portion of male sperm that is larger than the reference strain's sperm gets used for fertilization immediately and preferentially over the pre-existing male's smaller competitor sperm. Unexpectedly, we saw no increase in paternity when strains with larger sperm mated first in our double-mating assay (no P_1 _advantage). We suspect that the following scenario might explain this pattern. *C. elegans *lay ~9 eggs per hour at peak levels of oogenesis when they have high sperm availability [52], which corresponds well to the 20 - 40 fewer progeny of a given genotype over the 4 hr period that it is mated first relative to second in our sperm precedence assay, implying that our assay "missed" the first 20 - 40 progeny sired by the first male assayed. When a large-sperm male inseminates a female, she receives sperm that are variable with respect to size. If this large-sperm male is mated first, then the largest sperm will be used immediately (during the mating trial period; such eggs were discarded in our assay) and will have already fertilized oocytes by the time the smaller reference-strain sperm enter the reproductive tract. This would result in a situation where all sperm that remain in the reproductive tract after the mating trials will be of similar size and, therefore, of similar competitive ability. This overlap in the sperm size distribution could explain the pattern of 50% first-male paternity in our assay, even for strains that have larger sperm on average than males of the reference strain.

We observed that all P_1 _(early and late) and late P_2 _values for wild isolate-sired offspring did not differ from equal paternity (50%; Figure 6). Even given the model proposed above, it is unexpected that we do not observe higher paternity for second mated males (regardless of sperm size) in the late progeny, assuming an equal number of sperm are transferred by the two genotypes and that the first male's sperm is partially exhausted from fertilization during the mating trial itself. One pre-copulatory explanation for this finding is that the females might not facilitate mating as readily with second males. This idea is supported by the report that *C. elegans *hermaphrodites are less likely to mate if their reproductive tracts contain self-sperm [17], which likely extends to the case of male sperm being present in the reproductive tract. Males mate more easily with older hermaphrodites [35], that also will have fewer self-sperm in their reproductive tract, but the < 4-hour difference in age of females between mating trials is probably too small of a difference to reflect this age effect. Possible post-copulatory explanations include second-male sperm being flushed at a higher rate from the reproductive tract by egg passage, or, a higher rate of ejaculate ejection of second-male sperm - as observed in hermaphrodite-male sperm competition, such that hermaphrodites are more likely to eject male sperm when self-sperm are present [17]. Sperm age, however, is unlikely to have been an important factor, because previous work indicates that sperm age does not affect competitive ability in male-hermaphrodite sperm competition [24], and sperm in our experiment competed over several days but differed in age by only a matter of hours. In addition, this type of temporal variation of sperm use patterns has been seen in other systems [31].

Some studies have measured *C. elegans *male reproductive success by the ability of males to persist within androdioecious populations [29]. Although not statistically significant, given only 6 strains that could be included in correlation, Teotonio et al.'s [29] male maintenance ability metric showed the highest magnitude correlations with P2-late and with sperm size (Additional File 2), suggesting that these traits are worth further investigation for a role in the maintenance of males within *C. elegans *populations. Some of the male genotypes we assayed produce a mating plug [12,53]. Mating plugs affect re-mating rates in some taxa [13], but did not appear to affect P_2 _in this experiment. Our experimental design was such that the non-plugging reference strain PD4790 is the only strain that must mate following a mating plug deposit. It is formally possible that mating plugs retard re-mating ability more for some male genetic backgrounds, but that the reference strain (PD4790) males are largely unaffected by mating plugs. In this study, we focused mostly on sperm traits, but we expect that differences in other mating traits, such as time spent *in copula *or the incidence of sperm ejection by females, might also exhibit heritable variation contributing to differences in male reproductive success. Note that if females discriminate among male genetic backgrounds to cause differential sperm ejection, we would have expected significant differences among male genotypes in the assay of sperm transferred per ejaculate. Because we observed no such differences, it is unlikely that such a mechanism of female choice operated with the strains used in this study. An in-depth analysis of heritable variation in mating behaviors will help to fully dissect the relative importance of pre-mating, copulatory, and post-mating contributions to male reproductive success.

Because we use a reference strain to compare sperm precedence among wild isolates, we are unable to identify any non-transitive effects. Similarly, identical *fog-2 *female genotypes provide the arena for all sperm competition, so we cannot test for an effect of female genetic background on fertilization success. However, further investigation using sperm-depleted hermaphrodites from different strains or introgression of *fog-2 *(*q71*) into a variety of genetic backgrounds could provide valuable insight into variation in hermaphrodite and female mating traits. Indeed, Wegewitz et al. [30] showed recently that males are better at mating with strain CB4856 hermaphrodites than with hermaphrodites of the lab-adapted strain N2. In addition, CB4856 hermaphrodites have lower self-fecundity than do N2 hermaphrodites. They thus concluded that males are maintained more easily in populations of CB4856 both because the males are better at obtaining copulations and the hermaphrodites are worse at avoiding copulations than N2. Isogenic populations of strain CB4856 indeed retain males more readily than several other wild isolate and lab-adapted strains [29,54,55].

Mating rate also could positively affect the rate of sperm production, either as a consequence of selection or as a physiological byproduct. Alternatively, males with a high re-mating rate might transfer fewer sperm at each subsequent mating if the rate of sperm production is not modulated. Either of these scenarios would impact the resulting size distribution of sperm that a male produces, because larger, more-competitive sperm take longer to make.

It should be noted that mated *C. elegans *hermaphrodites have a decreased lifespan compared to non-mated hermaphrodites [56]. In our sperm precedence assay, individual female worms were exposed to a total of 16 male worms. This biased operational sex ratio, with corresponding male-induced harm to females, might have contributed to the mortality of 8 females prior to laying all of their eggs.

### *Understanding sperm size in *C. elegans

In the broader context of the genus, four explanations seem plausible for the small size of *C. elegans *sperm compared to obligately outcrossing species. First, tiny self-sperm might result from selection for rapid spermatogenesis in hermaphrodites because they cannot fertilize oocytes until sperm production is complete [27,57,58]. *C. elegans *hermaphrodite self-sperm are very small and have been proposed to be near the lower limit for sperm size given the constraints of sperm mobility [44]. This also is consistent with the minimal investment in male gametes by hermaphrodites that is expected from models of resource allocation in selfing organisms [59,60]. Second, the relatively small size of male *C. elegans *sperm compared to related gonochoristic species might be a byproduct of selection for small sperm in hermaphrodites via genetic correlation or pleiotropy. Third, male sperm size might have experienced relaxed selection on size when selfing hermaphroditism evolved, resulting in the evolution of smaller size. Developmental and/or mutational biases in the origin or evolution of sperm size also could generate small hermaphrodite and male sperm in the absence of strong countervailing selection [61]. Finally, selection may have favored decreased size of male sperm following the origin of selfing hermaphroditism, because male sperm no longer competed against other male sperm with any regularity and only had to be bigger than hermaphrodite sperm to ensure male fertilization success. At present, we cannot assess the relative likelihood of these alternatives.

### *The evolution of mating traits in *C. elegans *and its relatives*

Because *C. elegans *hermaphrodites mate so rarely, there is little reason for them to invest in large, competitive self-sperm. It might also be true that it is advantageous for hermaphrodites to outcross (masked recessive deleterious alleles, increased genetic variation, and other benefits of sex) with males when possible by allowing male sperm to win fertilization every time they mate. For example, some forms of stressful laboratory environments select for the maintenance of males in *C. elegans *[reviewed in [21,26]]. However, *C. elegans *lab and field data suggest that recombinant genotypes might not generally experience a selective advantage [62,63], which could indicate that selection for fast sperm production in hermaphrodites is a stronger selective force on sperm size and competitive ability than are benefits of outcrossing sex [64]. Indeed, changes in traits associated with outcrossing in *C. elegans *have been likened to the "selfing syndrome" described in plants [59]. Some of the traits related to this syndrome in hermaphrodites include a lack of mate searching behavior [65], their inability to produce potent pheromones to attract mates [36], and hermaphrodites' lack of mating facilitation behavior (particularly if they have self-sperm in their reproductive tract) [35]. In addition, hermaphrodites decrease cross-fertilization rates by ejecting male sperm post-mating [17,66]. *C. elegans *males are also less efficient at mating than their gonochoristic counterparts [35], and many wild strains (31%) have lost a functional version of a gene responsible for the ability to produce a copulatory plug after mating [12,53]. However, males of *C. elegans *are able to transfer sperm into different heterospecific partners [67] and are also attracted to the pheromones released by heterospecific females [36] which suggests that *C. elegans' *selfing syndrome manifests more strongly for hermaphrodite/female traits than for male traits.

Male-hermaphrodite sperm competition is likely the more important form of sperm competition in *C. elegans *because males are rare in nature [63,68] and are unlikely to encounter one another's sperm. Interestingly, *C. briggsae *(also androdioecious) exhibits similarly reduced male sperm size with even tinier hermaphrodite sperm [45]. However, male-male sperm competition surely is an important force in breeding system evolution in closely related gonochoristic species: males of outcrossing species have much larger sperm [45]. Here we have shown that *C. elegans *provides a tractable model to better understand the evolution of sperm competition patterns in *Caenorhabditis *species in general, which can shift toward direct tests as experimental tools are developed in other species, such as *C. remanei *[69]. It is critical to determine whether the relative importance of the various pre- and post-mating traits differs between species as a function of the intensity of male-male competition.

## Conclusions

Increased sperm size under sperm competition has been favored in a variety of taxa, despite the high variability in sperm form and function [6,7]. However, other traits also correlate with sperm size, such as fertilization priority [e.g., [70]] and preferential sperm storage [e.g., [71]], which complicates our understanding of the relative importance of sperm size and its general role in competitive ability. This study demonstrates that for *C. elegans *nematodes, it appears that sperm size and their rate of production represent dominant factors in deciding the success of male reproduction.

## Authors' contributions

RLM and ADC conceived and detailed the experimental design. JLK performed and developed the ejaculate size and mating assays. RLM prepared the manuscript and performed all other lab work. ADC directed this study. All authors discussed research strategies and development. All authors have read and approved this manuscript.

## Supplementary Material

Additional file 1 **Distribution of male sperm size**. Size distributions of male spermatids for each of 7 strains (diameter, cross-sectional area, and volume). Box-plots indicate median, inter-quartile range (points beyond whiskers are candidate outlier values). Click here for file

Additional file 2 **Pairwise correlations of male reproductive traits**. Pairwise correlations between average values of male reproductive traits for seven assayed isogenic strains. Click here for file
