# Supplementary material for: Heritable determinants of male fertilization success in the nematode Caenorhabditis elegans
Source: BMC Evol Biol. 2011 Apr 14;11:99. doi: 10.1186/1471-2148-11-99 (PMC3096603; doi:10.1186/1471-2148-11-99)

Additional File 1. Size distributions of male spermatids for each of 7 strains (diameter, cross-sectional area, and volume). Box-plots indicate median, inter-quartile range (points beyond whiskers are candidate outlier values).


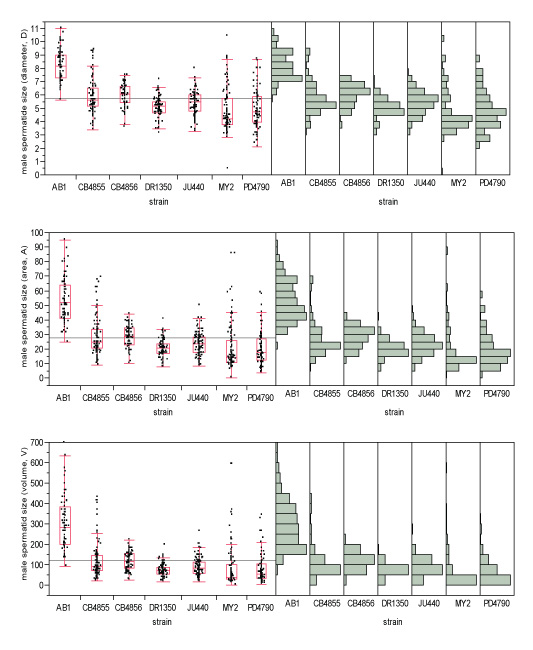

Supplement: Additional file 1 — Distribution of male sperm size. Size distributions of male spermatids for each of 7 strains (diameter, cross-sectional area, and volume). Box-plots indicate median, inter-quartile range (points beyond whiskers are candidate outlier values). [file 1471-2148-11-99-S1.DOCX]
