# Supplementary material for: Heritable determinants of male fertilization success in the nematode Caenorhabditis elegans
Source: BMC Evol Biol. 2011 Apr 14;11:99. doi: 10.1186/1471-2148-11-99 (PMC3096603; doi:10.1186/1471-2148-11-99)
Supplement: Additional file 2 — Pairwise correlations of male reproductive traits. Pairwise correlations between average values of male reproductive traits for seven assayed isogenic strains. [file 1471-2148-11-99-S2.DOCX]

Additional File 2. Pairwise correlations between average values of male reproductive traits for 7 assayed isogenic strains*.

|  | courtship ability ^a^ | mating ability  ^b^ | number of transferred sperm  ^c^ | sperm size ^d^ | sperm production rate ^e^ | P2 early | P2 late | P2 fertility ^f^ | male maintenance ^g^ |
| --- | --- | --- | --- | --- | --- | --- | --- | --- | --- |
| courtship ability |  | -0.20 | -0.18 | 0.39 | -0.79 | 0.61 | -0.14 | 0.43 | 0.26 |
| mating ability |  |  | -0.68 | -0.34 | 0.13 | -0.23 | -0.49 | -0.45 | -0.46 |
| number of sperm transferred |  | o |  | 0.04 | -0.07 | 0.18 | 0.14 | 0.21 | 0.20 |
| sperm size |  |  |  |  | -0.61 | 0.75 | 0.00 | 0.61 | 0.54 |
| sperm production rate | * |  |  |  |  | -0.93 | 0.29 | -0.54 | -0.14 |
| P2 early |  |  |  | o | ** |  | -0.04 | 0.75 | 0.03 |
| P2 late |  |  |  |  |  |  |  | 0.60 | -0.60 |
| P2_fertility |  |  |  |  |  | o |  |  | -0.20 |
| male maintenance |  |  |  |  |  |  |  |  |  |

* Spearman’s rank correlation above diagonal with significance level below diagonal (° 0.1 > P > 0.05, * P<0.05, ** P<0.01); ^a^ mean percent of observations with male-female contact and/or spicule insertion; ^b^ mean percent females inseminated in 9 h; ^c^ median number of sperm; ^d^ median diameter (μm); ^e^ mean number of sperm/min; ^f^ mean total number of progeny; ^g^ 6 strains only from Teotonio et al. [29]
